# Thermally-evaporated C_60_/Ag/C_60_ multilayer electrodes for semi-transparent perovskite photovoltaics and thin film heaters

**DOI:** 10.1080/14686996.2020.1780472

**Published:** 2020-07-22

**Authors:** Dong-Hyeok Choi, Hae-Jun Seok, Do-Hyung Kim, Su-Kyung Kim, Han-Ki Kim

**Affiliations:** a School of Advanced Materials Science and Engineering, Sungkyunkwan University, Suwon-si, Republic of Korea; b New & Renewable Energy Laboratory, Korea Electric Power Research Institute, Daejeon, Republic of Korea

**Keywords:** C_60_/Ag/C_60_ multilayer, thermal evaporation, flexibility, semi-transparent perovskite solar cells, transparent conductive electrodes, thin film heater, 201 Electronics / Semiconductor / TCOs, 209 Solar cell / Photovoltaics, 306 Thin film / Coatings

## Abstract

We investigated the characteristics of thermally evaporated fullerene (C_60_)/Ag/C_60_ (CAC) multilayer films for use in semi-transparent perovskite solar cells (PSCs) and thin-film heaters (TFHs). The top and bottom C_60_ layers and Ag interlayer were prepared using multi-source thermal evaporation, and the thickness of the Ag interlayer was investigated in detail for its effects on the resistivity, optical transmittance, and mechanical properties of the CAC electrodes. We used a figure-of-merit analysis to obtain a CAC electrode with a smooth surface morphology that exhibited a sheet resistance of 5.63 Ohm/square and an optical transmittance of 66.13% at a 550 nm wavelength. We conducted mechanical deformation tests to confirm that the thermally evaporated multilayer CAC electrode has a high durability, even after 10,000 times of inner and outer bending, rolling, and twisting due to the flexibility of the amorphous C_60_ and Ag interlayer. We evaluated the feasibility of using CAC electrodes for semi-transparent PSCs and TFHs. The semi-transparent PSC with 1.08 cm^2^ active area prepared with a transparent multilayer CAC cathode showed a power conversion efficiency (PCE) of 5.1%. Furthermore, flexible TFHs (2.5 × 2.5 cm^2^) fabricated on a thermally evaporated CAC electrode show a high saturation temperature of 116.6 C, even at a low input voltage of 4.5 V, due to a very low sheet resistance. Based on the performance of the PSCs and TFHs, we conclude that the thermally evaporated multilayer CAC electrode is promising for use as a transparent conductive electrode (TCE) for semi-transparent PSCs and TFHs, with characteristics comparable to sputtered TCEs.

## GRAPHICAL ABSTRACT

**Figure:**
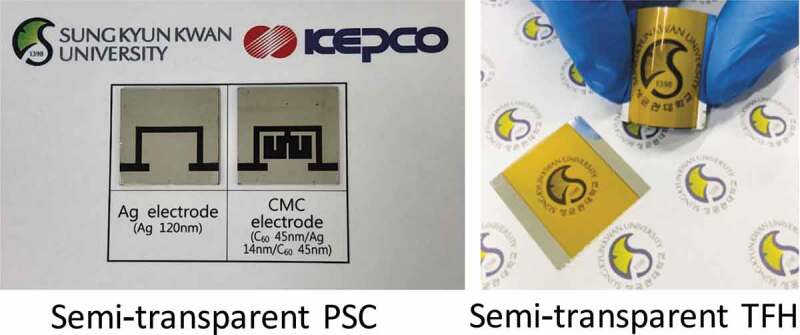

## 1. Introduction

Intelligent, next-generation buildings will employ promising technologies, including building automation systems, energy self-production systems, and functional energy-saving windows [1]. Functional windows have attracted attention because the number of windows on building exteriors has rapidly increased. These windows use semi-transparent photovoltaics (BIPVs) and transparent thin film heaters (TFHs) that have a critical role on the energy efficiency of the buildings.

Semi-transparent perovskite solar cells (PSCs) have been considered for use as BIPVs applied to glass windows, greenhouses, and rooftops due to their high power conversion efficiency (PCE) and thin-film fabrication process [2–4]. To fabricate highly efficient, semi-transparent PSCs, most layers comprising the PSCs, excluding the MAPbI_3_ photoactive layer, should be highly transparent. Considering the typical p-i-n planar PSCs architecture, the hole transport layer (HTL), active MAPbI_3_ layer, and electron transport layer (ETL) are sequentially coated on transparent anodes, and the device is completed with an opaque metal cathode, such as Ag and Cu. Therefore, to obtain semi-transparent PSCs, opaque metal cathodes should be replaced with transparent cathodes such as transparent conducting oxides (TCO), Ag nanowire, and carbon-based cathodes [5–7].

TCE has a great influence on the PCE, transmittance, color, stability, and mechanical properties in semi-transparent PSCs [8]. Over the past few years, TCEs such as TCOs, metals, carbon materials, and conducting polymer were extensively studied in semi-transparent PSCs [9–12]. In general, TCOs such as indium tin oxide (ITO), indium zinc oxide (IZO), aluminum-doped zinc oxide (AZO) are widely used as TCE due to their good transparency and conductivity. Because of excellent conductivity, various type of metals can be used as TCEs. However, since thick metal has low transparency, it is often used by a thin film type or a special structure for semi-transparent PSCs. In particular, dielectric/metal/dielectric (DMD) structure instead of thin metal film was usually introduced into semi-transparent PSCs [13–15]. For example, Gaspera et al. fabricated semi-transparent PSCs of 13.6% PCE with 7% AVT by engineering the thickness of MoOx/Au/MoOx [7]. In addition, Zhao et al. fabricated highly stable semi-transparent PSCs with SnOx/Ag/SnOx as the TCE [15]. Due to low-cost and roll-to-roll solution techniques, other forms of metal such as Ag NWs, Cu NWs, Ni mesh, and Ag mesh can be used as TCEs for semi-transparent PSCs. In addition, CNTs, graphene, and the conducting polymer (PEDOT:PSS) were also used as TCEs for semi-transparent PSCs.

Although the importance of transparent cathodes for semi-transparent PSCs has been emphasized, a detailed investigation of thermally evaporated hybrid cathodes is still lacking. Another important component in energy-saving functional windows are thin film heaters (TFHs) with high transparency and flexibility that can quickly and effectively remove frost or ice that may occur on the surface of the window. Since the electrical and optical characteristics of the transparent electrodes significantly affect the performance of the TFHs, such as the thermal response time and saturation temperature, it is essential to ensure high-performance transparent and flexible electrodes [1,16]. Various types of TCEs, including metal grid films, metal nanowires, conducting polymer, carbon nanotubes, and graphene, have been commonly used in photovoltaics, TFHs, and optoelectronic devices [17–23]. However, unlike sputtered TCO cathodes, solution-processed or transferred TCE electrodes cannot be applied to fabricate large functional windows due to their complicated processes and difficulty in coating a large area.

In this work, we present dielectric/metal/dielectric (DMD) transparent electrodes composed of a fullerene (C_60_)/Ag/C_60_ (CAC) multilayer grown via thermal evaporation for semi-transparent PSCs and TFHs. C_60_H_60_ (C_60_) generally called buckminsterfullerenes and has been widely used for various state-of-art devices such as field effect transistors, light-emitting diodes, chemical sensors, photodetectors, and photovoltaics because of the superior optical and electrical properties of C_60_ thin films [24,25]. Au or Ag is often used as the top metal electrode for carrier extraction in various electrical device [26]. Since it was mainly intended to investigate the change in the electrochemical properties of the electrode according to the C_60_ layer, silver, which is excellent in conductivity and widely used in DMD electrodes, was applied to the interlayer.

In the CAC multilayer, the Ag interlayer provides a conduction pathway to the CAC electrode, and two C_60_ layers were used to improve the optical transparency caused by the surface plasmonic resonance and destructive interference at the two Ag/C_60_ interfaces [27–29]. We evaluated various properties, such as the electrical, optical, and mechanical properties of the CAC multilayer with different Ag and C_60_ thicknesses to determine the optimal thickness. In addition, we demonstrated the flexibility of the CAC film coated on a colorless polyimide (CPI, Kolon CPI^TM^) film to use as flexible TCEs for TFHs. Furthermore, we fabricated semi-transparent PSCs and TFHs with an optimal multilayer CAC electrode and demonstrate the feasibility of using the CAC electrodes.

## 2. Experimental

### 2.1. Thermal evaporation of C_60_/Ag/C_60_ multilayer films

**Figure 1.**
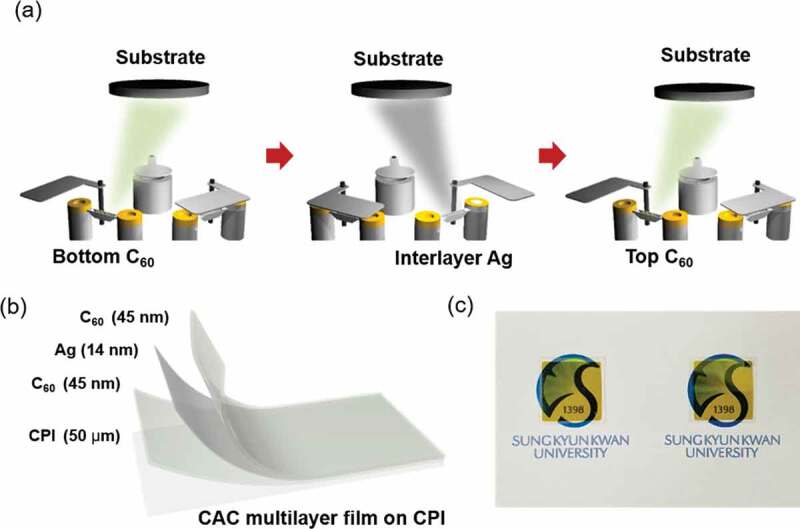
(a) Schematic illustration of consecutive thermal evaporation processes used to fabricate the CAC (45 nm/14 nm/45 nm) multilayer without breaking the vacuum. (b) CAC multilayer structure fabricated onto a CPI film (50 µm). (c) Photograph of the optimum thermally-evaporated CAC multilayer over the SKKU emblem.

A flexible, transparent CAC film was continuously deposited on a CPI film at room temperature using a thermal evaporation system (ITS-THS-3-50, I.T.S, Korea). A bottom C_60_ layer of various thicknesses was deposited under a vacuum of 1 × 10^−6^ Torr. Evaporation was conducted with a supplied current of 140 A, tool factor of 100%, Z-factor of 1.000, and rotational speed of 10 rpm for the loading plate. Since C_60_ have a relatively high melting point (~600°C), a high-quality C_60_ film can be obtained via thermal evaporation. After coating the C_60_ layer, an Ag layer of various thicknesses was consecutively deposited onto the bottom C_60_ layer with a supplied current of 80 A, tool factor of 100%, Z-factor of 0.529, and a rotational speed of 10 rpm for the loading plate. The C_60_ and Ag layers were deposited to a precise thickness using a thickness monitoring system installed in a thermal evaporator. After evaporation of the Ag layer, the top C_60_ was also deposited on an Ag layer with various thicknesses (Figure 1(a)). The evaporating and deposition conditions of two C_60_ layers were the same to form a symmetric CAC structure.

### 2.2. Characterization of C_60_/Ag/C_60_ multilayer electrodes

The electrical and optical properties of the CAC films were investigated with Hall measurements (HMS-4000AM, Ecopia, Korea) and with a UV/visible spectra analysis (UV 540 spectrometer, Unicam, Japan). The morphology of the CAC film was confirmed via Field Emission Scanning Electron Microscopy (FESEM, JSM-7600F, JEOL, Japan). The work function of the multilayer CAC was evaluated using Ultraviolet Photoelectron Spectroscopy (UPS, ESCALB-250Xi, Thermo Fisher Scientific, USA). The microstructure of the multilayer CAC was investigated with a High-Resolution Transmittance Electron Microscope (HRTEM, JEM-2100F, JEOL, Japan). The mechanical properties of the CAC film were analysed using a lab-made bending machine. The change in electrical resistance of the CAC film was measured with a 4-point probe station, and an optical microscope was mounted on the probe station to check for surface cracks. The bending test can be performed using two different approaches depending on the stress on the films. The outer bending test applied tensile stress and the inner bending test applied compressive stress to the CAC film. The bending radius is then calculated using

(1) $$Bending\;radius=\frac{L}{2\pi \sqrt{\frac{dL}{L}-\frac{\pi^{2}{h_{s}}^{2}}{12L^{2}}}R}$$

where *L, dL/L*, and *h_s_* denote the initial length, the applied strain, and the substrate thickness, respectively. The twisting durability of the CAC film was measured using a lab-made twisting test machine. The axis of rotation lies at the same height as the CAC film, and it is possible to rotate the substrate at an angle as low as 1°. The two RC servo motors rotate in opposite directions. The dynamic fatigue bending tests were carried out using a cyclic bending test machine at a frequency of 1 Hz over 10,000 cycles.

### 2.3. Fabrication and evaluation of PSCs with C_60_/Ag/C_60_ multilayer electrode

Semi-transparent p-i-n PSCs were fabricated to determine the feasibility of using the multilayered CAC cathode. The commercial ITO anode was cleaned using deionized water and ethanol in an ultrasonic bath for 20 min and was treated under UV/ozone for 30 min. Then, a hole transport layer, NiO was fabricated via spin coating at 4,000 rpm for 50 s, and the films were annealed at 285 C for 50 min. A 1.45 M Cs_0.175_FA_0.825_Pb(I_0.875_Br_0.125_)_3_ perovskite solution was prepared by mixing PbI_2_, PbBr_2_, FAI, FABr, and CsI in N,N-dimethylformamide (DMF) and dimethylsulfoxide (DMSO) (8:2 v/v) was deposited onto the HTL layer with a consecutive two-step spin-coating process at 500 and 5,000 rpm for 5 and 45 s, respectively. During the second spin-coating step, 400 µl of anhydrous chlorobenzene was dropped onto the substrate after 15 s, and then the substrate was dried at 100°C for 30 min. Finally, the CAC (45/14/45 nm) multilayer cathode was sequentially deposited using a thermal evaporation system. Dumbbell-shaped and square-shaped metal shadow masks were used to pattern the semi-transparent PSCs with an active area of 0.0464 cm^2^ and 1.08 cm^2^, respectively. J-V curves were obtained using a Ultraviolet solar simulator (SOL-UV-2, OMA, Newport, USA) with AM 1.5 G irradiation (100 mW cm^−2^) under ambient conditions.

### 2.4. Fabrication and evaluation of TFHs with C_60_/Ag/C_60_ multilayer electrode

To demonstrate the feasibility of the CAC film as a transparent electrode for TFHs, conventional thin-film heaters with size of 25 × 25 mm^2^ two-terminal side contacts were fabricated on the CAC multilayer electrode. For the terminal electrode, a 100 nm-thick Ag side contact electrode was sputtered onto the CAC multilayer. Both edge sides of the CAC films were coated with Ag paste and were connected with Cu tape, which acts as a contact electrode for the TFHs. DC power was applied to the TFHs through the Ag/Cu electrode located at the end of the film using a DC power supply (OPS 3010, ODA technology, Korea). The temperature of TFHs was measured directly using a thermocouple mounted on the surfaces of the transparent and flexible TFHs and measured indirectly using an Infrared thermal imager (A35sc, FLIR, USA).

## 3. Results and discussion

Figure 1(a) shows the schematics of the fabrication process for the CAC film obtained via thermal evaporation with multi-tungsten boats. In the thermal evaporation process, two C_60_ layers and an Ag interlayer are deposited on 50 μm-thick CPI substrates without breaking the vacuum. The symmetric C_60_ layers were evaporated using the tungsten boat on the left and the Ag layer was deposited using the tungsten boat on the right. The structure of the CAC film (45/14/45 nm) on the CPI substrate is as shown in Figure 1(b). The bottom C_60_ layer acts as an interfacial adhesive layer between the C_60_ and CPI film. The Ag interlayer plays an important role as the main current path in the CAC multilayer. In addition, the top C_60_ layer acts as a dielectric layer to implement antireflection in the CAC multilayer. The optimized CAC film exhibited a sheet resistance of 5.63 Ω/square, resistivity of 5.8 × 10^−5^ Ω-cm, and transmittance of 66.13% at 550 nm wavelength. Figure 1(c) is photograph of the yellowish CAC film. The semi-transparency of the CAC film makes it possible to see the emblem of SKKU behind the sample.

**Figure 2.**
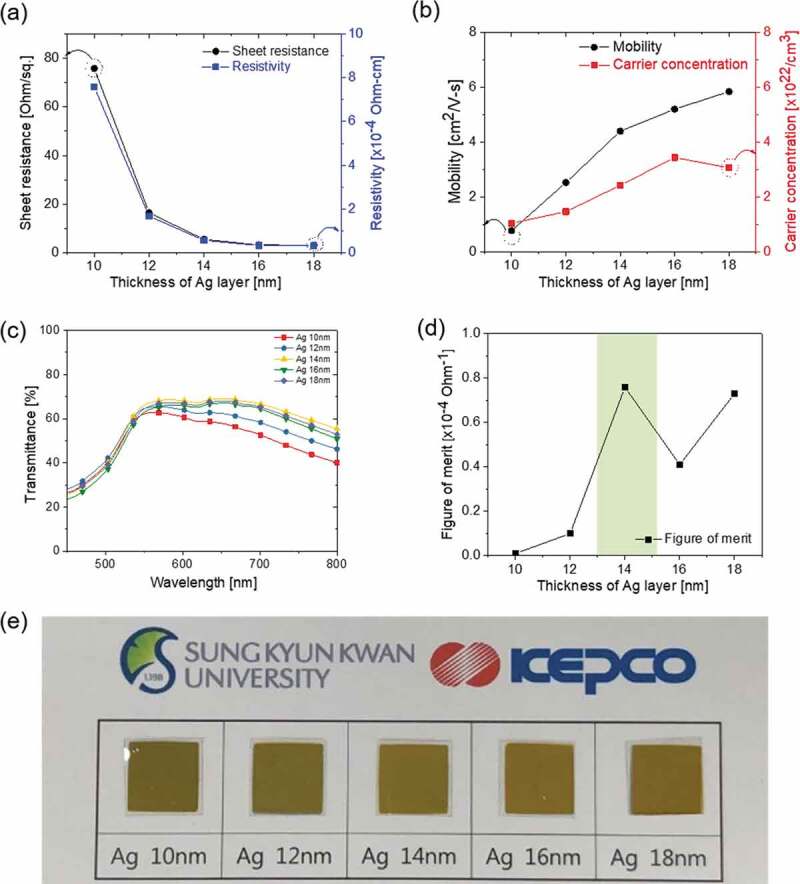
(a) Sheet resistance and resistivity and (b) mobility and carrier concentration of the CAC film with increasing Ag interlayer thickness at a fixed C_60_ thickness of 45 nm. (c) Optical transmittance of the CAC film in the visible wavelength region. (d) Figure of merit (FOM) values of the CAC film versus the thickness of the Ag interlayer. (e) The color and transparency of the CAC film with increasing Ag interlayer thickness.

Figure 2(a) and (b) exhibit the Hall measurement results of the CAC films as the thickness of the Ag layer increased (10, 12, 14, 16, 18 nm) with a constant symmetric C_60_ thickness of 45 nm. As discussed in our previous work [1,30–33], the optical transparency and electrical conductivity of the DMD layers considerably depend on the thickness of the multiple thin layers, particularly the metal interlayer. Therefore, the reported metal interlayer thickness for the optimized DMD electrode ranged from 8 to 14 nm [29,34–37]. The interlayer shape, such as islands or a continuous layer, is well known to be critically dependent on the thickness of the metal layer. The CAC film with a 10 nm-thick Ag layer exhibited a significantly high sheet resistance of 75.80 Ohm/square and a resistivity of 7.58 × 10^−4^ Ohm·cm due to randomly connected Ag islands, as shown in Figure 2(a). When the Ag layer thickness increased from 10 nm to 18 nm, the sheet resistance of the CAC film was confirmed to be significantly reduced, indicating the formation of layered Ag. As discussed by Indluru et al., the Ag layer provided a main current path in the CAC film, and the total conductivity is affected by the connectivity of the metal interlayer [38]. Bender et al. also observed an increase in the AgCu thickness in the DMD structure film that decreases the specific resistivities of the overall multilayer [39]. The decreased resistance properties of the CAC film can be explained by the increase in the carrier concentration and mobility, using the following relation:

(2) $$\rho =\frac{1}{ne\mu }$$

where ρ is the resistivity, n is the number of charge carriers, *e* is the charge of the carrier, and µ is the mobility. As shown in Figure 2(b), with an increase in the Ag interlayer thickness, the carrier concentration and mobility of the CAC film simultaneously increased from 1.05 × 10^22^ to 3.07 × 10^22^ cm^−3^ and 0.78 to 5.84 cm^2^/V-s. The increased carrier concentration and mobility of the CAC structure could be attributed to an improved connectivity of the Ag layer with an increasing Ag thickness [40]. The optical transmittance of the CAC films according to the Ag interlayer thickness is shown in Figure 2(c). The CAC film exhibited a high transmittance caused by the antireflection of the DMD structure [41–44]. In particular, the CAC film with a 14 nm-thick Ag layer exhibited the highest transmittance of 66.13% at a wavelength of 550 nm. Even though it has a similar thicknesses of an oxide-metal-oxide multilayer prepared by sputtering, the CAC films showed a lower optical transmittance due to the dielectric constant in the range of 3.61 to 4.4 of C_60_ [45]. However, with the thickness of the Ag metal layer exceeding 16 nm, the transmittance of the CAC film decreased. Therefore, it is important to find the optimum top/bottom C_60_ and Ag thickness to achieve a high optical transmittance. We obtained and evaluated the figure of merit (FOM = T^10^/R_sh_) values calculated from the sheet resistance (R_sh_) and optical transmittance (T). The CAC multilayer with 14 nm-thick Ag interlayer showed the best FOM value of 7.66 × 10^−5^ Ohm^−1^, with the highest transmittance of 66.13% even though it had higher sheet resistance at 5.63 Ohm/square than the other CAC samples with thicker Ag interlayer, as shown in Figure 2(d).

Through the results above of the CAC film, we confirmed that the optimal thickness of the Ag interlayer was 14 nm to obtain high-performance multilayer CAC electrodes, expressed as a shadow region in Figure 2(d). Table 1 summarizes the electrical and optical properties of a CAC film according to the Ag interlayer thickness.

**Table 1.** Electrical and optical properties of the CAC electrodes with increasing Ag interlayer thickness at a fixed C_60_ thickness of 45 nm.

Ag thickness [nm]	Sheet resistance [ohm/square]	Resistivity [✕10^−4^ ohm-cm]	Mobility [cm^2^/V-s]	Carrier Con. [✕10^22^/cm^3^]	Trans. _Average_ [%]
10	75.80	7.58	0.78	1.05	38.33
12	16.50	1.68	2.53	1.47	41.86
14	5.63	0.58	4.40	2.43	46.08
16	3.29	0.34	5.20	3.44	41.08
18	3.23	0.34	5.84	3.07	43.42

**Figure 3.**
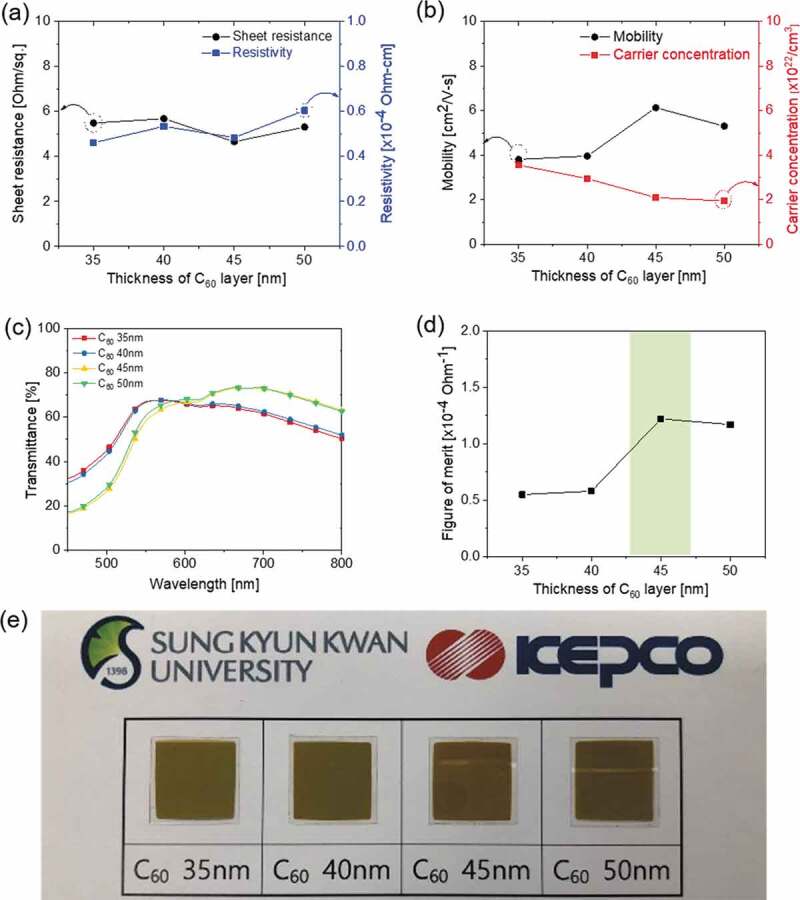
(a) Sheet resistance and resistivity and (b) mobility and carrier concentration of CAC film with an increasing C_60_ layer thickness at a fixed Ag interlayer thickness of 14 nm. (c) Optical transmittance of the CAC film in the visible wavelength region. (d) Figure of merit (FOM) values of the CAC film versus the thickness of the C_60_ layers. (e) The color and transparency of the CAC film with increasing C_60_ interlayer thickness.

Figure 3(a) and (b) exhibit the results of the Hall measurement for the CAC film with an increasing thickness of symmetric C_60_ layers at a constant Ag thickness of 14 nm. It should be noted that all CAC films exhibited a similar sheet resistance (4.66–5.68 Ohm/square) at a fixed Ag thickness independently of the symmetric C_60_ layer thicknesses since the main current path in the CAC is the Ag interlayer. However, the resistivity of the multilayer CAC tended to increase as the thicknesses of the top and bottom C_60_ layers increased. The resistivity of the CAC film increased due to the high resistivity of the symmetric C_60_ layers, as shown in Figure 3(a). The increased resistivity could be attributed to the reduced carrier concentration, as shown in Figure 3(b). The carrier concentration of the CAC film decreased as the overall thickness increased. As the symmetric C_60_ layer thickness increased at a fixed Ag thickness, the carrier concentration transferred from the Ag layer decreased, which increased the resistivity of the CAC film. Figure 3(c) exhibited the transmittance of the CAC film with an increase in the symmetric C_60_ thicknesses. The optical transmittance increased as the symmetric C_60_ layer thickness increased from 35 to 50 nm. The CAC multilayer with 50 nm-thick symmetric C_60_ layers was shown to have the highest transmittance of 73.36% at a wavelength of 690 nm. However, as shown in Figure S1, as the thickness of the symmetric C_60_ layer further increases, the optical transmittance tended to decrease. As the symmetric C_60_ thickness increased, the absorption band of the CAC film shifted to a long wavelength, as shown in Figure S1. In the CAC multilayer, optical transmittance increases due to suppression of surface plasmon resonance and destructive interference of reflected light from the interface and the surface when the refractive index and thickness of the dielectric layer and the reflective metal interlayer are adequate [27–29]. However, if the thickness of the C_60_ layer is over 50 nm, the suppression of surface plasmon resonance and the destructive interference effect of reflected light are reduced, thereby reducing optical transmittance. Accordingly, it was found that the optimum thickness of the C_60_ layer was between 35 nm and 50 nm.

Through the sheet resistance and average transmittance for the CAC film, the FOM values according to the thickness of the symmetric C_60_ layer were obtained, as shown in Figure 3(d). The CAC film with a 45 nm C_60_ layer exhibiting the best FOM value of 1.22 × 10^−4^ Ohm^−1^ caused a low sheet resistance of 4.66 Ohm/square and a high transmittance of 73.36% at a wavelength of 690 nm. The electrical and optical properties of the CAC film as a function of the symmetric C_60_ layer thickness are summarized in Table S1. Based on the electrical and optical properties of the CAC films with different C_60_ and Ag thicknesses in Figures 2 and 3, we determined the optimal thickness of the multilayer CAC to be 45/14/45 nm.

Figure S2 exhibited FESEM images of the CAC film as the Ag interlayer thickness increased from 10 to 18 nm. Regardless of the Ag interlayer thickness, all CAC films showed similar surface morphology and features due to the amorphous C_60_ layer on top. Even at an Ag thickness of 10 nm, the CAC exhibited smooth and very flat surface morphology because the top C_60_ layer fully covered the Ag interlayer. Consequentially, thermally evaporated CAC films showed a smooth morphology and featureless surface without protrusion or surface defects, regardless of the Ag layer thickness. Figure S3 exhibits the surface morphology of the CAC film with an increase in C_60_ thickness from 35 to 50 nm. All CAC films also exhibited a similar morphology in spite of the different top C_60_ thickness.

**Figure 4.**
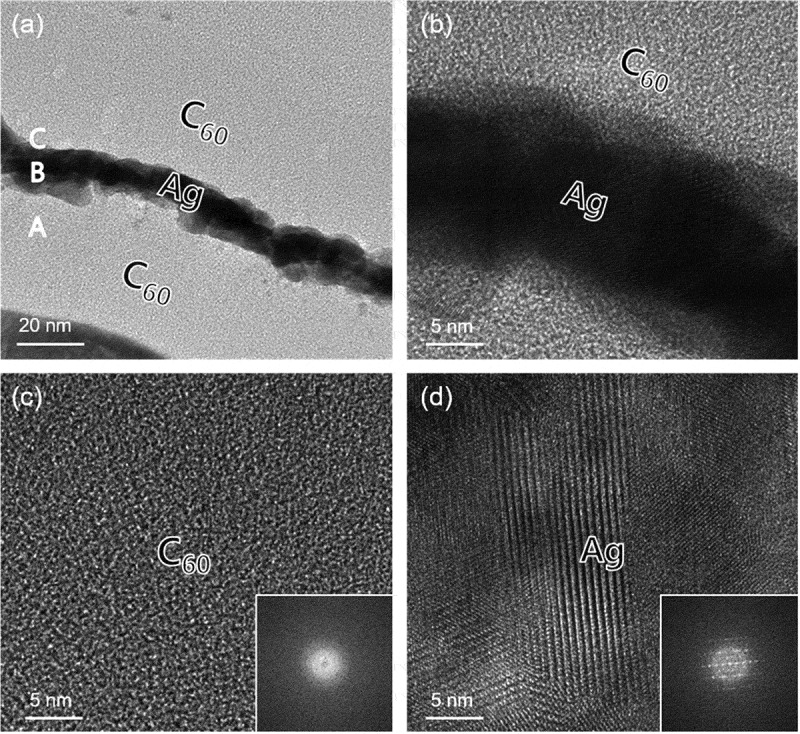
(a) Cross-sectional TEM image of the thermally evaporated C_60_/Ag/C_60_ multilayer film on a CPI substrate. Enlarged TEM images obtained from A, B, and C in the cross-sectional image with an inset of FFT patterns, (b) C_60_/Ag interface region, (c) bottom C_60_ region, and (d) Ag interlayer region, respectively.

HRTEM analysis was conducted to confirm the interface and microstructure of the CAC film. Figure 4(a) shows a TEM image of the CAC (45/14/45 nm) film on a CPI substrate. The symmetric CAC structure was confirmed in the TEM image indicating a well-controlled thickness of the C_60_ and Ag layers. The bright and dark regions in the TEM image are the top/bottom C_60_ and Ag interlayer. The TEM image definitely exhibited well-defined symmetric C_60_ layers and Ag interlayer without interfacial layers. The enlarged image of the interface of the Ag interlayer and top C_60_ layer in Figure 4(b) definitely showed that the dark Ag interlayer was well-contacted with the top and bottom C_60_ layer. Due to continuous thermal evaporation, there is no interfacial layer between the bright C_60_ and dark Ag interlayer. The uniform contrast of the bottom C_60_ layer in Figure 4(c) indicates that the thermally evaporated C_60_ layers had a completely amorphous structure. The diffuse fast Fourier transform (FFT) pattern in inset of Figure 4(c) also confirmed the amorphous structure of the thermally evaporated C_60_ layer. However, the enlarged TEM image in Figure 4(d) obtained from the Ag interlayer showed a well-developed crystalline structure. The thermally evaporated Ag interlayer with a well-distinctive crystalline structure could act as main current path in CAC multilayer electrodes. The strong spot in the FFT pattern (inset of Figure 4(d)) also confirms the polycrystalline structure of the Ag interlayer in the CAC multilayer.

**Figure 5.**
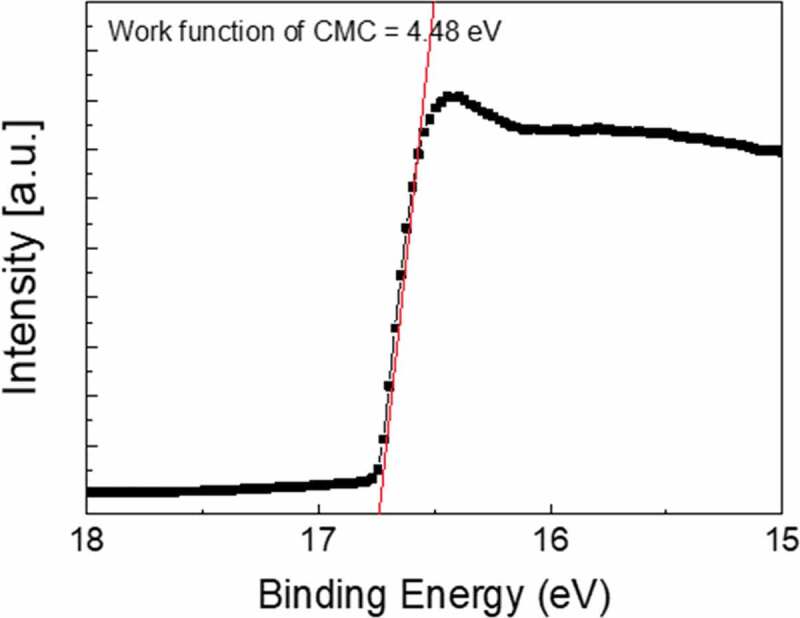
Work function of an optimized C_60_/Ag/C_60_ multilayer electrode obtained from a UPS analysis.

As shown in Figure 5, the work function (Φ) of the optimized CAC film was evaluated by UPS. The work function of the CAC film can be defined as Φ = *hv* – *ΔE*, where *hv* is the energy of photon (21.22 eV) and *ΔE* is obtained from the binding energy between the secondary electron emission cut-off edge in the UPS spectra [46,47]. As a result of the UPS measurement, the work function of the CAC multilayer (4.48 eV) was lower than that of the ITO film (4.7–4.8 eV) and a metallic Ag metal layer (4.7 eV) [48–50]. Therefore, the lower work function of the CAC film showed the feasibility of the thermally evaporated CAC electrode as an effective cathode for perovskite solar cells.

**Figure 6.**
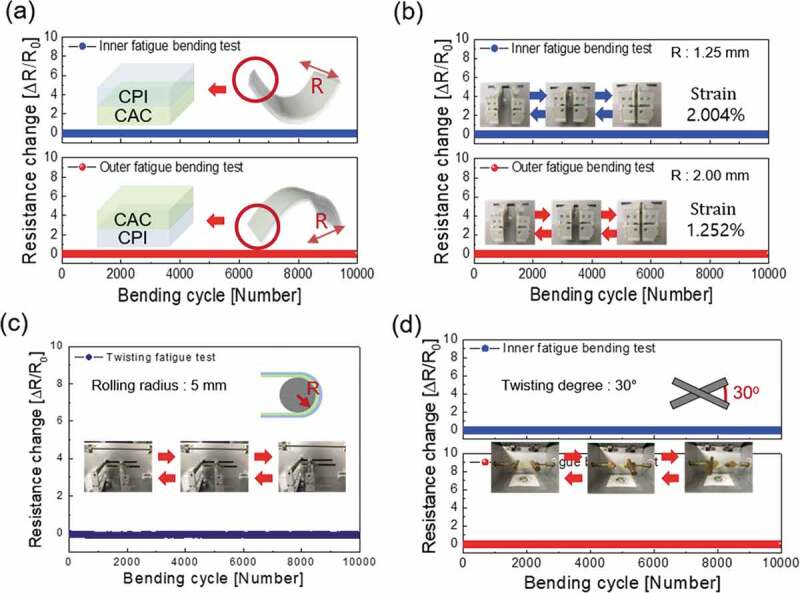
(a) Resistance changes of the outer/inner bending radius tests of multilayer CAC film on CPI (50 µm) in accordance with the bending radius (b) Resistance changes of the inner/outer bending fatigue tests of the multilayer CAC film on CPI (50 µm) in accordance to increase bending cycles. The bending radius of inner bending fatigue tests is 1.25 mm, bending radius of outer bending fatigue test is 2.00 mm. (c) The changes in resistance of the rolling and (d) twisting fatigue tests of the CAC film on CPI (50 µm) in accordance to an increase in bending cycles. The rolling radius of the rolling fatigue tests is 5 mm, and the maximum twisting angle of the twisting fatigue test is 30°.

To investigate the mechanical flexibility and durability of the optimized CAC (45/14/45 nm) electrode, we carried out inner/outer bending fatigue, rolling fatigue, and twisting fatigue tests for 10,000 cycles using lab-designed test systems. Figure 6(a) exhibited the results of the outer/inner bending radius tests of the CAC film on CPI (50 µm) in accordance to the bending radii. For the CAC samples that had been tested for bending, the change in resistance (ΔR = R – R_0_) as a function of decreasing bending radius was evaluated, where R_0_ is the initial resistance of the sample, and R is the resistance under CAC/CPI bending. As shown in Figure 6(a), the CAC film showed a constant resistance until the bending radius reached 1.0 mm. The peak strain for a CAC electrode with a decreasing bending radius can be obtained using the following equation:

(3) $$Strain=\frac{d_{CACmultilayer}+d_{CPI}}{2R}\times 100\%$$

**Figure 7.**
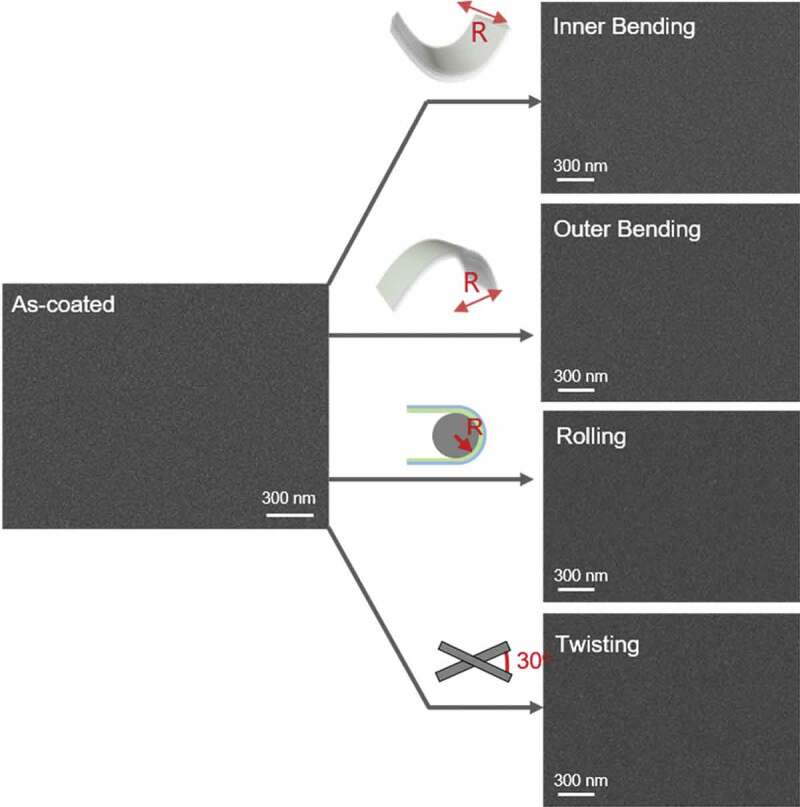
Surface FESEM images of (left) the as-coated CAC film, CAC film after an inner fatigue test, outer fatigue test, twisting fatigue test, and parallel CF thin film after the rolling fatigue test.

Here, R is the bending radius and $d_{CACmultilayer}andd_{CPI}$.are the thicknesses of the CAC multilayer (104 nm) and CPI film (50 µm), respectively [43]. Bending of the to 1.0 mm radius resulted in a peak strain of 2.505%. Therefore, we could confirm that the electrical properties were maintained even in harsh conditions of a 1.0 mm bending radius. Regardless of the outer or inner bending mode, the multilayer CAC film exhibited a small critical bending radius of 1.0 mm, as described in the top and bottom panel of Figure 6(a). The dynamic bending fatigue tests for the Multilayer CAC film with a constant inner and outer bending radius of 1.25 mm radius and 2.0 mm are shown in Figure 6(b), respectively. No change in the resistance (ΔR) was confirmed during bending cycles of the dynamic inner and outer bending fatigue tests, demonstrating an excellent durability and flexibility of the CAC film. Figure 6(c) and (d) show the changes in resistance of the rolling and twisting fatigue tests of the CAC film with an increase in the bending cycles. The rolling radius is fixed as 5 mm, and the twisting angle is also fixed at 30°. The inset in Figure 6(c) and (d) showed rolling and twisting steps during fatigue tests. The results showed that the CAC film had a constant resistance during the rolling and twisting fatigue test. Therefore, the electrical properties of the CAC film were sustained even after harsh bending fatigue tests. Furthermore, to verify the mechanical durability of the CAC electrode, the surface morphology of the CAC electrode was examined via FESEM before and after 10,000 cycles of mechanical fatigue tests. As shown in Figure 7, even after various harsh fatigue tests, the CAC multilayer electrode showed an identical surface morphology with as-deposited CAC sample without any defects, such as surface cracks, delamination, and so on.

**Figure 8.**
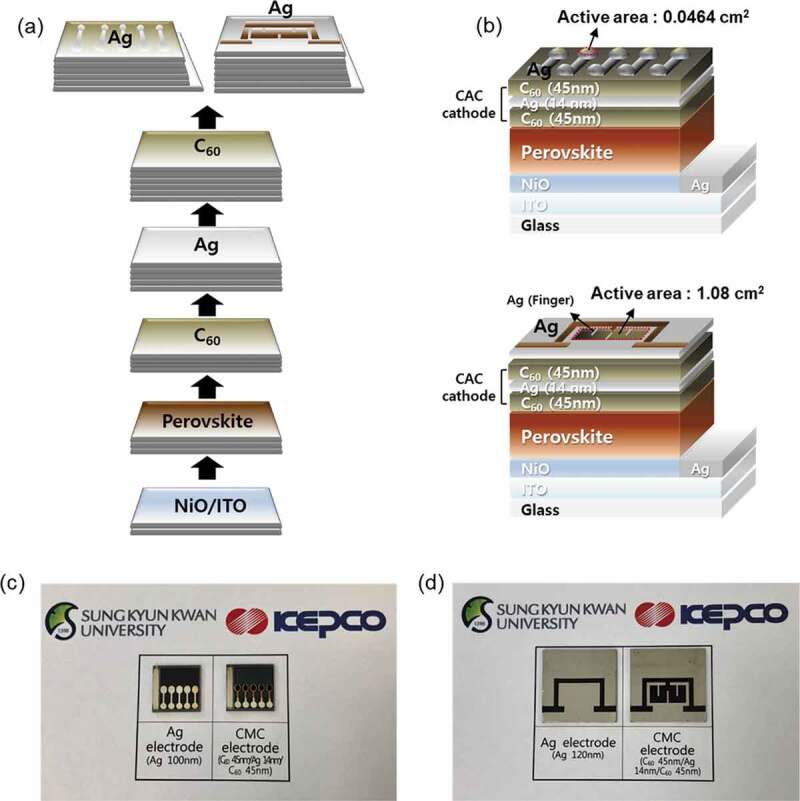
(a) Schematics of the fabrication process and (b) device structures of the perovskite solar cell with a CAC electrode. (c) and (d) Pictures of PSC devices with top Ag (Ref.) and CAC electrodes.

To evaluate the feasibility of using the optimized CAC (45/14/45 nm) film as a transparent cathode, semi-transparent p-i-n PSCs were fabricated and evaluated. The fabrication process, architecture, and picture of the PSC devices (active area 0.0464 cm^2^) are presented in Figure 8. As shown in Figure 8(a), the Cs_0.175_FA_0.825_Pb(I_0.875_Br_0.125_)_3_ perovskite layer (~500 nm thick) was first deposited on the ITO/glass coated with a thin (~30 nm) p-type NiO layer. The CAC (45/14/45 nm) cathode was then deposited sequentially using a thermal evaporation system by using dumbbell- and square-shaped metal masks with an active area of 0.0464 cm^2^ and 1.08 cm^2^, respectively (Figure 8(b)).

For comparison, we fabricated reference PSCs deposited with Ag (120 nm) as a cathode on ETL layer. Figure 8(c) and (d) show pictures of semi-transparent and opaque PSCs with CAC and Ag cathode. It is clearly shown that the PSCs with CAC cathode demonstrate optical transparency. This indicates that the CAC film is a prospective cathode for semi-transparent PSCs because it has higher transparency than a typical thick metal cathode.

**Figure 9.**
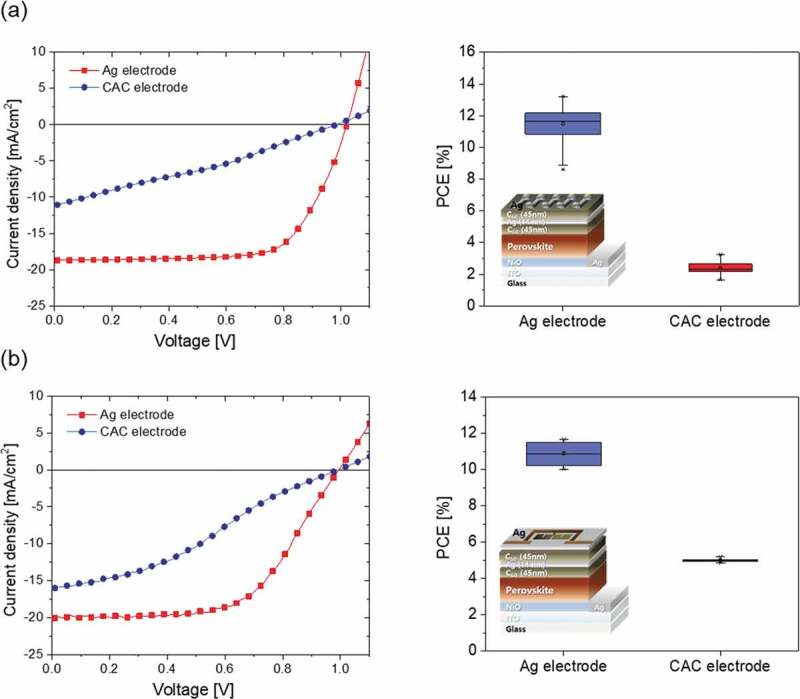
(a) J–V curves and photovoltaic performance statistics of the top Ag and CAC-based PSC devices with an active area of 0.0464 cm^2^. (b) J–V curves and photovoltaic performance statistics of top Ag and CAC-based PSC devices with an active area of 1.08 cm^2^.

Figure 9(a) and Table 2 present the performance of PSCs with 0.0464 cm^2^ active area prepared with CAC multilayer and reference Ag cathode, respectively. The J-V curve of the PSCs with CAC cathode yields an open circuit voltage (V_oc_) of 0.98 V, a short circuit current (J_sc_) of 11.1 mA/cm^2^, a fill factor (FF) of 29.7%, and a PCE of 3.2%. Reference devices prepared using a thick and opaque Ag cathode (120 nm) showed a PCE above 13.2%. In the p-i-n structure semi-transparent PSCs, the cathode plays a role of transferring the generated electrons to an external conductor, and has an important effect on the performance of the PSCs. Accordingly, in order to fabricate a PSCs having high performance, it is necessary to secure an electrode having good electrical properties such as sheet resistance, carrier concentration, and mobility. In this study, although the electrical properties of the CAC film of the optimum thickness are excellent, the performance of the PSCs with CAC cathode was low.

**Table 2.** Photovoltaic performance of cells (0.0464 cm^2^) with Ag and CAC electrodes.

Parameter	Voc [V]	Jsc [mA/cm^2^]	FF [%]	PCE [%]
Ag electrode	1.01	18.7	69.2	13.2
CAC electrode	0.98	11.1	29.7	3.2

A much lower PCE of semi-transparent PSC with a CAC cathode is related to the low value of J_sc_ and the fill factor, suggesting that the top C_60_ layer interferes with the smooth extraction of electrons from the ETL. A similar effect has been recently confirmed for PSCs fabricated using a DMD electrode [7]. In particular, the thicker C_60_ layer influenced the charge extraction properties of PSC due to the limited conductivity of C_60_ layer, and as a result, although the CAC electrode shows low sheet resistance and high transmittance, it is estimated that the series resistance of the entire PSCs increases. If the thickness of Ag is increased, the carrier concentration and mobility will be increased, and metallic Ag layer will be able to easily extract electrons in CAC film. Figure 9(b) and Table 3 exhibit the performance of PSCs with a 1.08 cm^2^ active area prepared with the CAC cathode. The PSCs with the CAC cathode showed the following cell performance: Voc of 0.99 V, Jsc of 16.0 mA/cm^2^, fill factor of 32.6%, and PCE of 5.1%.

**Table 3.** Photovoltaic performance of cells (1.08 cm^2^) with Ag and CAC electrodes.

Parameter	Voc [V]	Jsc [mA/cm^2^]	FF [%]	PCE [%]
Ag electrode	0.99	19.9	58.7	11.6
CAC electrode	0.99	16.0	32.6	5.1

The reference devices prepared using an opaque Ag cathode exhibited a PCE above 11.6%. The advantage of the CAC multilayer structure is that it has a high optical transparency compared to the thick metal. Even if the thick (~100 nm) Ag layer shows opaque characteristics, the thin (~20 nm) Ag layer exists between symmetric C_60_ dielectric layers, and each layer thickness can be adjusted to increase the transparency of the electrode through destructive interference in the visible range. Although the PCE of the semi-transparent PSCs fabricated with the CAC cathode is lower than that of PSCs with thick metal cathode, we convince that further optimization of thickness of the Ag interlayer and C_60_ dielectric layer and fabrication process could increase the PCE of the semi-transparent PSCs. As soon as we improve the performance of perovskite solar cell with CAC electrode, we will report the data in this journal in detail.

**Figure 10.**
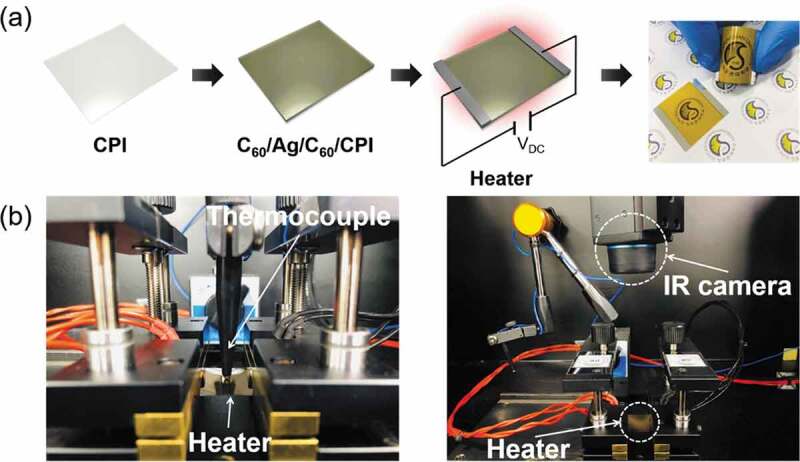
(a) Schematic structure and picture of flexible and transparent thin film heaters fabricated with thermally evaporated C_60_/Ag/C_60_ multilayer electrodes. (b) Thermocouple and IR camera used in heating test of CAC-based TFHs.

As another prospective application of the CAC film, we fabricated TFHs with a size of 2.5 × 2.5 cm^2^ using a two Ag contact electrode composition. Figure 10(a) exhibits the configuration and images of the TFHs with CAC electrodes. DC power was applied to TFHs through the Ag electrode located at the end of the film, and the temperature profile of TFHs was measured by a thermocouple installed on the surface of TFH. Moreover, an infrared (IR) thermal image was taken with an IR thermometer. Figure 10(b) shows the method to measure the temperature using a thermocouple and an IR thermal imaging camera.

**Figure 11.**
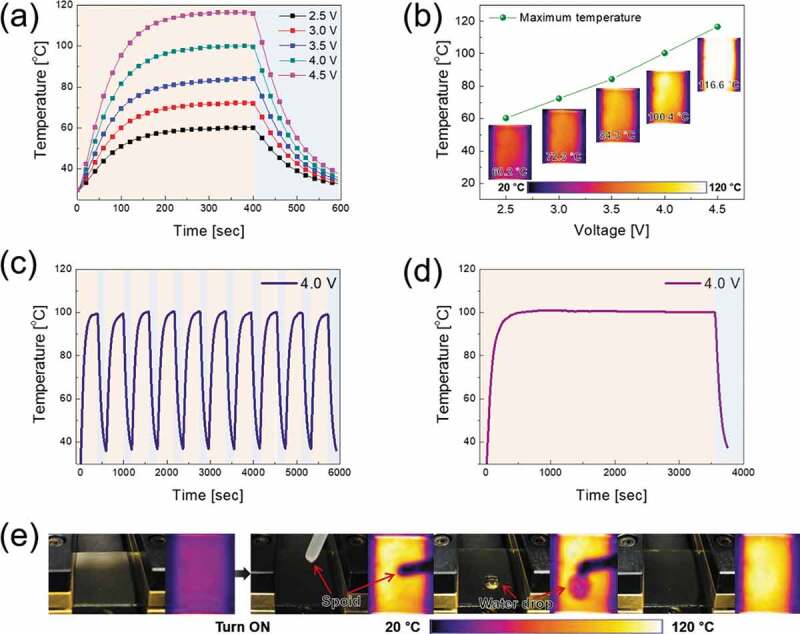
(a) and (b) Temperature profiles of CAC-based TFHs under operation at different input voltages. The inset shows IR images at the saturation temperature. (c) Repeated heating and cooling cycles of CAC-based TFHs for 10 times. (d) Temperature profile when a constant DC voltage was supplied to the electrode for 1 h. (e) Images of the water droplet test on heated TFHs with a C_60_/Ag/C_60_ multilayer electrode.

Figure 11(a) exhibits the temperature profiles of the TFHs fabricated on the optimum CAC film as transparent electrodes. The CAC-based TFHs reached 100°C with a very low power caused by its low sheet resistance. The time-dependent temperature profiles of the CAC multilayer-based TFH indicate that the performance of the CAC multilayer-based heater is dependent on the sheet resistance of the transparent electrodes. Due to the low sheet resistance (5.63 Ω/square), the TFH with the CAC multilayer showed a uniform saturation temperature distribution. However, the TFH with the ITO electrode (at the DC voltage of 3.9 V) fabricated in our previous study showed a lower saturation temperature (45.7°C) and non-uniform temperature distribution due to its higher sheet resistance (40.25 Ω/square) [31].

When power was applied to TFHs, the temperature rose rapidly and saturated. When the voltage was increased from 2.5 to 4.5 V, the saturation temperature of the TFH reached 116.6°C as shown in Figure 11(b). The high saturation temperature of the CAC-based TFHs with a lower sheet resistance indicates that electrical energy at the electrode is effectively converted to Joule heating. By Joule’s law, the sheet resistance of TCE for TFHs can be correlated with the generated heat [21,51,52]. The power (P) supplied to the TFHs during the heating time (t) generated heat (∆Q_g_):

(4) $$\Delta Qg=P\Delta t\;=\;\frac{V^{2}}{R}\Delta t\;=\;Q_{conv}$$

Here, V is the DC voltage between electrodes, R is the resistance of the TFHs, and Q_conv_ is the convective heat.

Through the above equation, TFHs with a low sheet resistance at a constant voltage can be seen to generate a higher temperature. Therefore, a low resistance TCE is important to fabricate TFHs that can operate at lower voltages. To investigate the durability of TFHs, repeated heating and cooling tests were performed for 10 cycles, as shown in Figure 11(c). When a DC voltage of 4.0 V was applied and removed, the CAC-based TFHs exhibited the same heating and cooling temperature profile. During the 10 cycles, the TFHs achieved a saturation temperature near 100 C. Moreover, as shown in Figure 11(d), when a DC voltage of 4.0 V was applied to the TFHs for 1 h, the TFHs retained a temperature of 100 C. To ensure the CAC-based TFHs effectively remove water, a water droplet test was carried out on TFHs. The images of the water droplet test and the IR thermometer readings of the TFHs with a temperature near 100°C are shown in Figure 11(e). Immediately after the DC voltage of 4.0 V was applied to the TFHs, Joule heating and convection reached a dynamic balance, and a saturation temperature of 100°C was achieved. Therefore, the water droplet evaporated instantly due to the heat generated from the CAC-based TFHs. The results of temperature profile of the TFHs showed that the CAC film is a prospective electrode for high-performance TFHs.

## 4. Conclusions

In conclusion, we investigated various properties of the CAC films for semi-transparent PSCs and TFHs. The correlation between the thickness of the symmetric C_60_ layers and Ag layer was investigated to find the optimal thickness of each layer according to the electrical, optical, and mechanical properties of the CAC film. According to the figure of merit, we fabricated a CAC electrode with a low sheet resistance of 5.63 Ohm/square and high transmittance of 66.13% at 550 nm wavelength with good surface morphology. Furthermore, the CAC electrode exhibited superb mechanical flexibility in the bending fatigue, twisting fatigue and rolling fatigue tests. Besides, the low work function (4.48 eV) of the CAC film demonstrated the possibility of using the CAC electrode as a semi-transparent cathode for PSCs. Semi-transparent PSCs were fabricated and evaluated with CAC cathode to assess the feasibility of using the optimized CAC (45/14/45 nm) electrode. Semi-transparent PSCs with 1.08 and 0.0464 cm^2^ active area prepared with CAC multilayer cathode showed a PCE of 5.1% and 3.2%, respectively. Although the semi-transparent PSCs with CAC cathode showed lower PCE than opaque PSCs with thick Ag cathode, the successful operation of semi-transparent PSCs indicates the feasibility of the CAC multilayer electrode as a cathode. As a result of the performance of the CAC-based TFHs, the CAC film was found to be a prospective electrode for TFHs. The performance of the TFH was evaluated according to the sheet resistance of the CAC electrodes to confirm the important role of the TCEs for high-performance TFHs. Consequently, as a result of the performance of the PSCs and TFHs, we confirmed that the optimized CAC film is a prospective electrode for semi-transparent PSCs and TFHs for next-generation smart windows.
